# Role of miRNAs in Sigmoid Colon Cancer: A Search for Potential Biomarkers

**DOI:** 10.3390/cancers12113311

**Published:** 2020-11-10

**Authors:** Diego Marques, Layse Raynara Ferreira-Costa, Lorenna Larissa Ferreira-Costa, Ana Beatriz Bezerra-Oliveira, Romualdo da Silva Correa, Carlos Cesar de Oliveira Ramos, Tatiana Vinasco-Sandoval, Katia de Paiva Lopes, Ricardo Assunção Vialle, Amanda Ferreira Vidal, Vivian Nogueira Silbiger, Ândrea Ribeiro-dos-Santos

**Affiliations:** 1 Laboratório de Genética Humana e Médica, Universidade Federal do Pará, Av. Augusto Corrêa, 01, Guamá, Belém 66.075-110, Brazil; diegomarquescs@outlook.com (D.M.); gtvinascos@gmail.com (T.V.-S.); katiapaiva@ufpa.br (K.d.P.L.); vialle@ufpa.br (R.A.V.); amandaferreiravidal@gmail.com (A.F.V.); 2 Laboratório de Bioanálise e Biotecnologia Molecular, Universidade Federal do Rio Grande do Norte, Av. Nilo Peçanha, 620, Petrópolis, Natal 59012-300, Brazil; laysecostta@hotmail.com (L.R.F.-C.); lorennacostta@hotmail.com (L.L.F.-C.); anabboliveira@hotmail.com (A.B.B.-O.); 3 Departamento de Cirurgia Oncológica, Liga Norte Riograndense Contra o Câncer, R. Mário Negócio, 2267, Quintas, Natal 59040-000, Brazil; romualdocorrea@uol.com.br; 4 Laboratório de Patologia e Citopatologia, Liga Norte Riograndense Contra o Câncer, R. Mário Negócio, 2267, Quintas, Natal 59040-000, Brazil; ramosccesar@hotmail.com; 5 Programa de Pós-Graduação em Genética e Biologia Molecular, Universidade Federal do Pará, Av. Augusto Corrêa, 01, Guamá, Belém 66.075-110, Brazil; 6 Núcleo de Pesquisas em Oncologia, Universidade Federal do Pará, R. dos Mundurucus, 4487, Guamá, Belém 66073-000, Brazil

**Keywords:** biomarkers, field-effect, colorectal cancer, miRNome

## Abstract

**Simple Summary:**

Understanding the microRNAs’ role in cancer is challenging, because, in the most cases, the only tissues available are the tumor and its counterpart, the adjacent-to-tumor tissue. Indeed, this scenario could affect our analyses in the cancer field, including to colorectal cancer. Using systematic criteria to select the healthy individuals and sigmoid colon cancer cases, we found that adjacent-to-tumor tissue already has molecular alterations in relation to healthy tissue and to tumor tissue as well. In addition, all miRNAs found showed to be involved in the carcinogenic process, according to the gene enrichment analysis. We suggested that future cancer studies should consider using these three tissues in their analysis, as well as the creation of a database of healthy individuals’ miRNA expression profiles. Using these three tissues, we could better understand the role of adjacent-to-tissue in cancer and other several questions related with clinical aspects.

**Abstract:**

The aberrant expression of microRNAs in known to play a crucial role in carcinogenesis. Here, we evaluated the miRNA expression profile of sigmoid colon cancer (SCC) compared to adjacent-to-tumor (ADJ) and sigmoid colon healthy (SCH) tissues obtained from colon biopsy extracted from Brazilian patients. Comparisons were performed between each group separately, considering as significant *p*-values < 0.05 and |Log_2_(Fold-Change)| > 2. We found 20 differentially expressed miRNAs (DEmiRNAs) in all comparisons, two of which were shared between SCC vs. ADJ and SCC vs. SCH. We used miRTarBase, and miRTargetLink to identify target-genes of the differentially expressed miRNAs, and DAVID and REACTOME databases for gene enrichment analysis. We also used TCGA and GTEx databases to build miRNA-gene regulatory networks and check for the reproducibility in our results. As findings, in addition to previously known miRNAs associated with colorectal cancer, we identified three potential novel biomarkers. We showed that the three types of colon tissue could be clearly distinguished using a panel composed by the 20 DEmiRNAs. Additionally, we found enriched pathways related to the carcinogenic process in which miRNA could be involved, indicating that adjacent-to-tumor tissues may be already altered and cannot be considered as healthy tissues. Overall, we expect that these findings may help in the search for biomarkers to prevent cancer progression or, at least, allow its early detection, however, more studies are needed to confirm our results.

## 1. Introduction

Colorectal cancer (CRC) is the third most common cancer and the cause of a considerable number of deaths worldwide [1]. Despite the continuous progress in diagnostic and therapeutic methods [2,3], Brazil is the sixth and seventh in incidence and mortality, respectively [1].

CRC is not a single type of tumor and its pathogenesis depends on the anatomic location [4], differing between the colon (which is subdivided into seven parts: cecum, ascending, hepatic flexure, transverse, splenic flexure, descending, and sigmoid) and rectum [5]. Depending on the anatomical location and stage, CRC has a different molecular biological characteristic, which results in distinct clinical practices [6,7].

Advances in molecular biology have demonstrated that subsequent genetic and epigenetic alterations are required to initiate the carcinogenesis process, and drive the progression of adenomas to carcinomas in sporadic and inherited forms of CRC [8,9]. This process can take decades to escape the multiple cellular regulatory layers and fully develop [8,9].

To date, there are three significant pathways associated with CRC pathogenesis—chromosomal and microsatellite stability, and CpG island methylation [9,10]. However, non-coding RNAs have also been described as key in this process, especially the microRNAs [11]. These biomarkers—for instance—may identify patients who are most likely to benefit from an individual treatment [6,11,12,13].

MicroRNAs (miRNAs) are small non-coding RNAs (~22 nucleotides) involved in the regulation of mRNA translation [14]. Each miRNA has the potential to interact with more than one mRNA, which in turn may be suppressed by several miRNAs [15]. Moreover, miRNAs are involved in multiple cellular functions, including those related to malignant transformation, such as angiogenesis, cell growth, genomic stability, and inflammatory response [15,16].

Recent studies have related miRNAs as potential biomarkers to improve the diagnosis and prognosis; and have analyzed its application to cancer treatment, as reviewed by Liu and Chen [17]. However, currently, we are only scratching the surface; a significant amount of research is still required to (a) generate a complete picture of miRNA expression and its clinical relevance in CRC; (b) gain knowledge of potential targets, and their molecular effects; and (c) determine whether they will fulfill their promise as biomarkers [18].

Keeping this necessity in mind, in recent years, our research group has been exploring the role of these molecules in several aspects, including the characterization of the miRNA expression profile in the human stomach [19,20]; and their dysregulation in gastric and oral cancer [21,22,23,24,25,26,27]. Additionally, we highlight their importance to the field cancerization process in gastric cancer [23,26,27], which comprehends a group of cells in the adjacent to tumor mass that can be altered and be further ahead in the evolutionary path towards cancer [28].

Thus, considering that each colon portion has different molecular biological features—implying a specific clinical practice to CRC [29,30]—and the field cancerization theory, in the present study, we evaluate the global expression profile of miRNAs in sigmoid colon cancer (SCC) and measure the differences with sigmoid colon healthy (SCH) and adjacent-to-tumor (ADJ) tissues.

## 2. Materials and Methods

### 2.1. Biological Material

**Figure 1:**
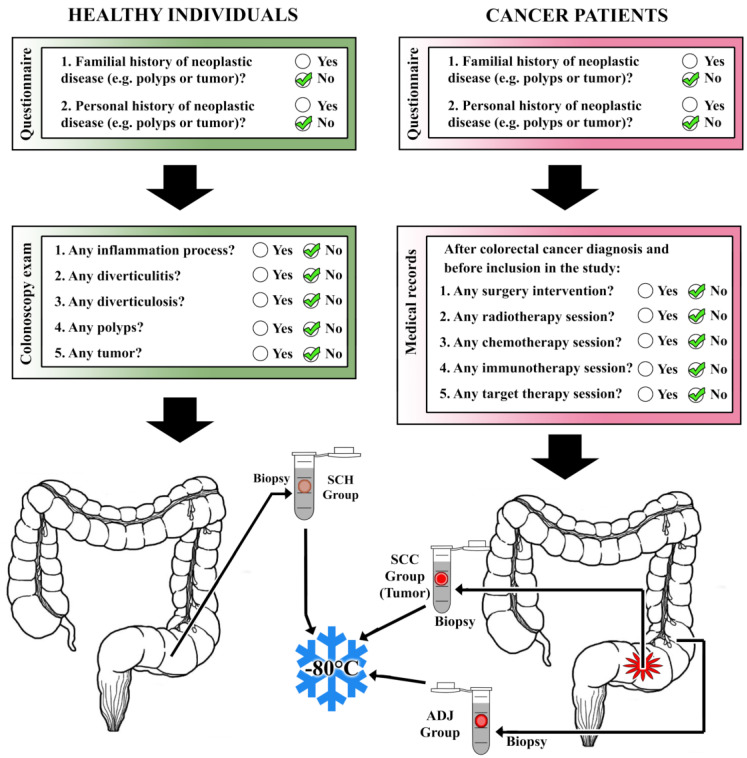
Flowchart of casuistic selection.

Our samples are divided in two groups: healthy individuals and cancer patients. Healthy individuals were submitted to the colonoscopy exam. The biopsy was collected without any alteration (e.g., inflammation process, diverticulitis, diverticulosis, polyps, or tumor). Additionally, none of them had a personal or familial history of neoplastic diseases (polyps or tumors, for instance). Regarding the cancer patients, we collected a serial biopsy from those who had not had a prior diagnosis of cancer and no previous clinical intervention (surgery, radiotherapy, chemotherapy, immunotherapy, or target therapy). These criteria are schematized and illustrated in Figure 1.

In the present study we included a total of 21 fresh samples of colon tissues. Of these, there were seven biopsies of sigmoid colon cancer tumor (SCC), seven biopsies from the adjacent-to-tumor tissue (ADJ, approximately 5~10 cm from tumor marge, avoiding necrotic tissue), and seven biopsies from the sigmoid colon of healthy individuals (SCH).

The SCH tissues were obtained from healthy volunteers from the Centro de Diagnóstico de Imagens do Hospital Universitário Onofre Lopes (Rio Grande do Norte—Natal). Regarding the cancer patients, samples were obtained from patients treated at the Liga Norte Riograndense Contra o Câncer (Liga; Rio Grande do Norte—Natal). Immediately after collection, all samples were immersed in RNAlater^®^ Stabilization Solution (Ambion; Life Technologies, Carlsbad, CA, USA) and stored at −80 °C until analysis.

Clinical data were collected from the pathological reports from the Liga’s department of pathology (Table 1). Histopathological analysis of the tumor fragments was performed according to the International Agency for Research on Cancer [31].

### 2.2. Ethics Statement

This study was reviewed and approved by the Ethics Committee of the Liga Norte Riograndense Contra o Câncer (Protocol N°: 36185514.2.0000.5293). All study participants or their legal guardian provided informed written consent following the Helsinki Declaration of 2013, the Nuremberg Code, in compliance with the National Health Council’s Research Guidelines Involving Human Beings (Res CNS 466/12).

### 2.3. RNA Extraction, Small RNA Library Preparation, and Sequencing

Total RNA was extracted using TRIzol^®^ reagent (Thermo Fisher Scientific). After isolation, total RNA was stored at −80 °C until further analysis. Total RNA was quality tested using the 2200 TapeStation Instrument (Agilent Technologies, Santa Clara, CA, USA). Samples with an RNA integrity number ≥ 5 were sequenced. For small RNA-seq, 1 μg of total RNA per sample was used for library preparation using TruSeq Small RNA Sample Prep Kits (Illumina, San Diego, CA, USA). A 4-nM library pool comprising all samples was sequenced using a MiSeq Reagent Kit with v3-150 cycles on a MiSeq System (Illumina, San Diego, CA, USA). The raw sequencing reads of all libraries were deposited at the European Nucleotide Archive (PRJEB37027).

### 2.4. Bioinformatics Analysis

Raw reads were preprocessed using Trimmomatic (v.0.36) [32] to remove adaptors, low-quality bases, and reads with <16 nucleotides. We used the STAR aligner [33] to map the reads to the human genome reference (GRCH37). We quantified mature miRNA sequencing according to the miRbase human annotation (v20) using the HTSeq [34].

Differential expression analysis was performed using the DESeq2 package in the R software [35], filtering miRNA with <10 reads. Comparison between [a] Sigmoid Colon Cancer (SCC) vs. Sigmoid Colon Healthy (SCH); [b] Adjacent to Sigmoid Colon Cancer (ADJ) vs. SCH; and [c] SCC vs. ADJ samples were made separately. FDR adjusted *p*-value < 0.05 and |Log_2_(Fold-Change)| > 2 were considered statistically significant.

For graphical analysis, expression data were normalized using Median-Ratio Normalization (MRN). Heatmaps were used for hierarchical clustering visualization of the differentially expressed miRNAs (DEmiRNAs); a DAPC (Discriminant Analysis of Principal Components) was constructed to infer the number of clusters of the related samples [36]. All graphical analyses were performed using the R statistical platform v3.6.2 (http://www.r-project.org/).

### 2.5. Identification of the miRNAs’ Target Genes

We used two online tools to identify the target genes of DEmiRNA: [i] miRTarBase v.7.0 and [ii] miRTargetLink Human. We considered as target genes the overlapping results between these databases.

**Figure 2:**
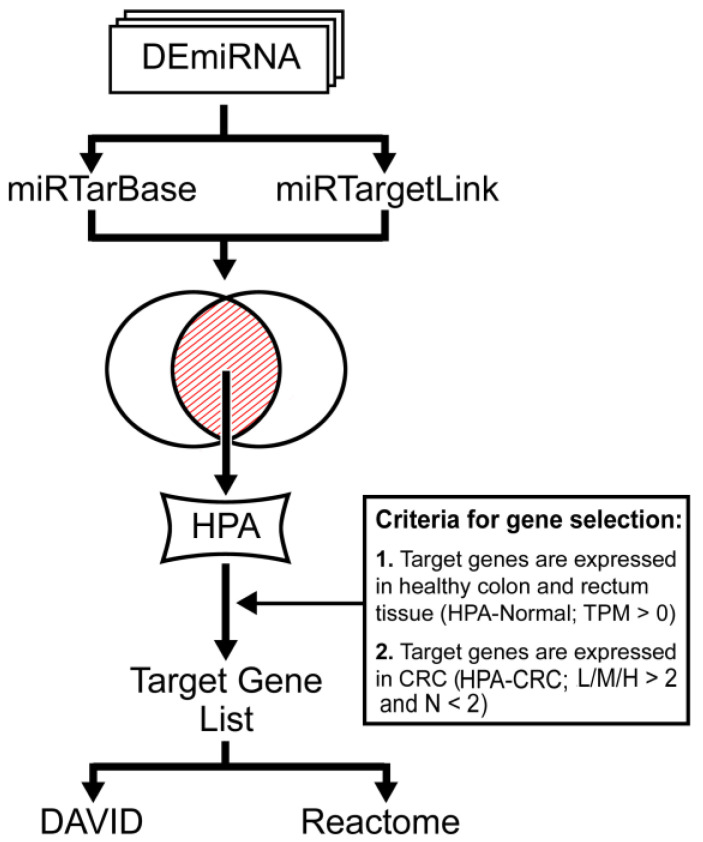
Flowchart of the target genes selection. For normal tissues (HPA-Normal), the HPA quantifies the expression as transcripts per million units (TPM). We considered target genes in normal tissues those with TPM > 0. For tumor tissues (HPA-CRC), the HPA classifies protein expression according to staining intensity (negative—N; low—L; moderate—M; or high—H). We considered target genes in tumor tissues, those that have lower than two negative intensity cases, and at least one of others with staining intensity supported by more than two cases.

To identify the target genes that are expressed in colon tissues, we used the “hpar” Bioconductor package to access Human Protein Atlas (HPA) databases [37]. We selected gene expression on normal (colon and rectum) and tumoral tissue, according to the HPA database. The criteria are schematized and illustrated in Figure 2.

The identified target genes were submitted to functional annotation and enrichment in KEGG pathways using DAVID Bioinformatics Resources v.6.8 online tool [38,39]. Then, we used the REACTOME Pathway Database to explore the over-represented pathways and topology. Cytoscape v.3.7.2 was used to create a network of DEmiRNAs and DEGenes. The interaction between potentially targeted genes was performed using STRING [40], considering only interactions: curated from database or text-mining, experimentally determined, co-occurrent, and co-expressed. Disconnected nodes were hidden from the network view.

**Figure 3:**
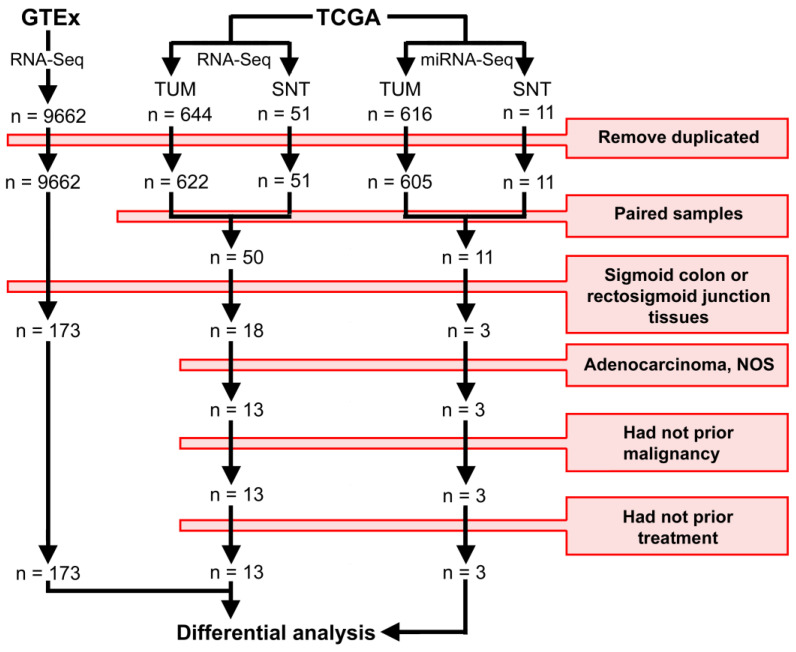
Data mining workflow. The GTEx data were downloaded using the recount v.1.14.0 package. The TCGA data were downloaded using the TCGAbiolinks v.2.16.1 package. The differential analyses were performed using the DESeq2 package. Transcripts with FDR adjusted *p*-value < 0.05 and |Log_2_(Fold-Change)| > 2 were considered statistically significant. TUM = Primary Tumor. SNT = Solid Normal Tissue. The red box indicates the filters applied during casuistic selection.

We used The National Cancer Institute Genomic Data Commons (The Cancer Genome Atlas, TCGA) and GTEx (Genotype-Tissue Expression) databases to obtain transcriptome data from the healthy, tumor, and adjacent-to-tumor tissues to perform differential expression analysis. The criteria for casuistic selection are detailed in Figure 3. Clinical features of TCGA participants were extracted from cBioPortal for Cancer Genomics (Table 2).

## 3. Results

After quality control, alignment, and transcript quantification, the average number of mapped reads per sample was 53,021, varying from 11,620 to 372,268. From a list of 2576 known miRNAs, 666 were detected, with raw read count ≥1 in at least one sample. Of them, 160 miRNAs presented more than ten reads on average and were selected for further investigation. Considering this threshold, the most abundant miRNA was hsa-miR-143-3p (responsible for ~33% of the expression), followed by *hsa-miRNA-192-5p*, *hsa-miRNA-10a-5p*, and *hsa-miR-10b-5p*.

### Differentially Expressed miRNAs Analysis

**Figure 4:**
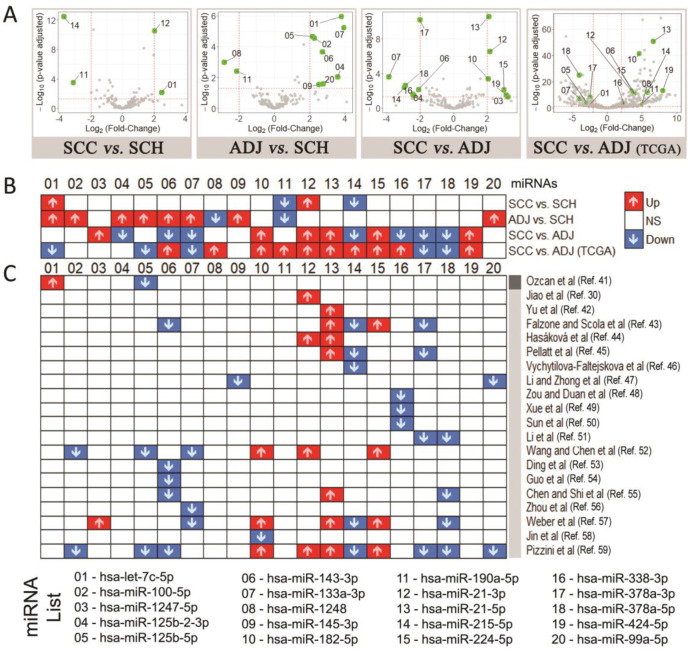
Overview of the differential miRNA expression analysis. (**A**) Volcano plot showing the differential expression analysis, (**B**) an overview of 20 differential expressed miRNAs in this study, and (**C**) a comparison of these 20 miRNAs with results of previous studies with CRC patients. These studies were annotated as Dark-grey and Light-grey according to tissue considered as reference. Dark-grey = this study used a biopsy of a healthy subject as a reference against Colorectal Cancer (CRC). Light-grey = these studies used adjacent-tumor tissue as the reference against CRC. Note: Sigmoid Colon Cancer (SCC); Sigmoid Colon Healthy (SCH); Adjacent to Sigmoid Colon Cancer (ADJ). Up = Upregulated. Down = Downregulated. NS = Not-significant or Not-evaluated.

Differential expression analyses were performed following these three comparisons: [a] SCC vs. SCH (four DEmiRNAs); [b] ADJ vs. SCH (ten DEmiRNAs); and [c] SCC vs. ADJ (13 DEmiRNAs), resulting in a total of 20 DEmiRNAs – these results are presented in Figure 4A. As shown in Figure 4B, some DEmiRNAs are shared between the models, while others are exclusive: two DEmiRNAs are shared by SCC vs. ADJ and SCC vs. SCH (*hsa-miR-21-3p* and *hsa-miR-215-5p*), and five DEmiRNAs were found only in ADJ vs. SCH (*hsa-miR-100-5p*, *hsa-miR-1248*, *hsa-miR-145b* and *hsa-miR-99a-5p*).

In Figure 4C, we compared our results with previous studies. We found that most of our DEmiRNAs were previously described in CRC, except for *hsa-miR-125b-2-3p*, *hsa-miR-1248*, *hsa-miR-190a-5p*, and *hsa-miR-424-5p*, indicating that these miRNAs could be potential novel biomarkers. We also performed a differential analysis using TCGA miRNA-Seq data for comparison – data mining workflow is detailed in Figure 3. Overall, we found nine DEmiRNAs in common and with the same expression pattern between our comparison of SCC vs. ADJ and TCGA (Figure 4B).

**Figure 5:**
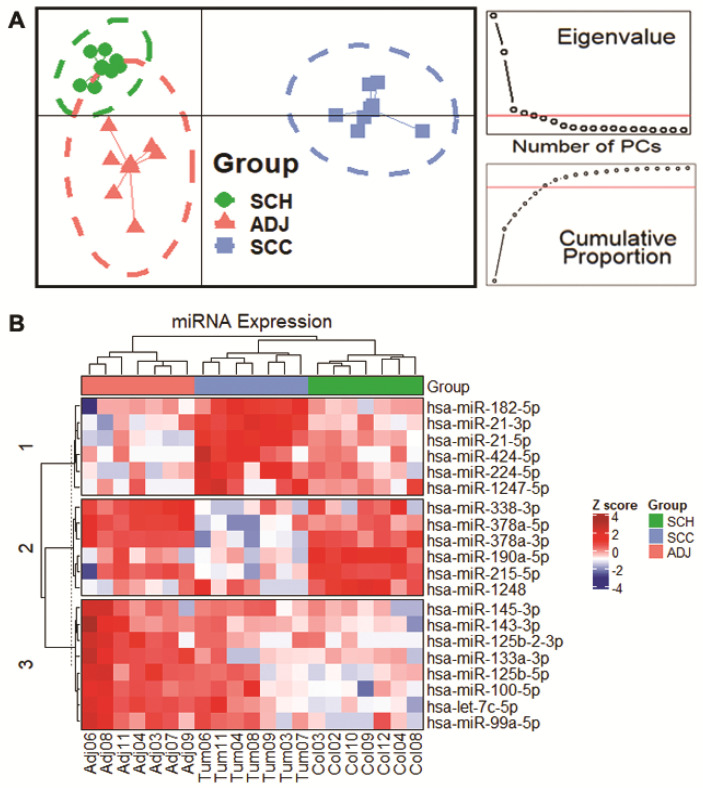
A clustering-based approach for efficient identification of microRNA combinatorial biomarkers. (**A**) Discriminant Analysis of Principal Components (DAPC) and (**B**) Heatmap plot of all differentially expressed miRNAs, clustered by Euclidean distance. In DAPC, the individuals are represented as dots and the groups as inertia ellipses. The axes represent the first two Linear Discriminants (LD). The dashed line represents the 95% confidence interval. Principal Components (PC) eigenvalues retained in the dimension-reduction step of the analysis are displayed inset, the Kaiser criterion threshold is represented as a red line. Additionally, we used the cumulative proportion to assess the total amount of variance that the consecutive principal components explain 90% of cases (red line). In the heatmap, there are three miRNAs clusters. Cluster 1—differences between tumor expression from their counterpart (adjacent tissue). Cluster 2—discern tumor expression from healthy tissue. Cluster 3—discriminate adjacent-tumor expression from healthy tissue. Note: Sigmoid Colon Cancer (SCC); Sigmoid Colon Healthy (SCH); Adjacent to Sigmoid Colon Cancer (ADJ).

Our set of 20 DEmiRNAs was used to perform a DPAC (Figure 5A) and a heatmap (Figure 5B). Both analyses show evidence that these miRNAs can distinguish each group of samples (SCH, ADJ, and SCC), suggesting its high specificity. It also indicates that ADJ tissue has different expression from healthy and tumor tissues and cannot be considered as normal tissue, reinforcing the field cancerization theory in CRC.

**Figure 6:**
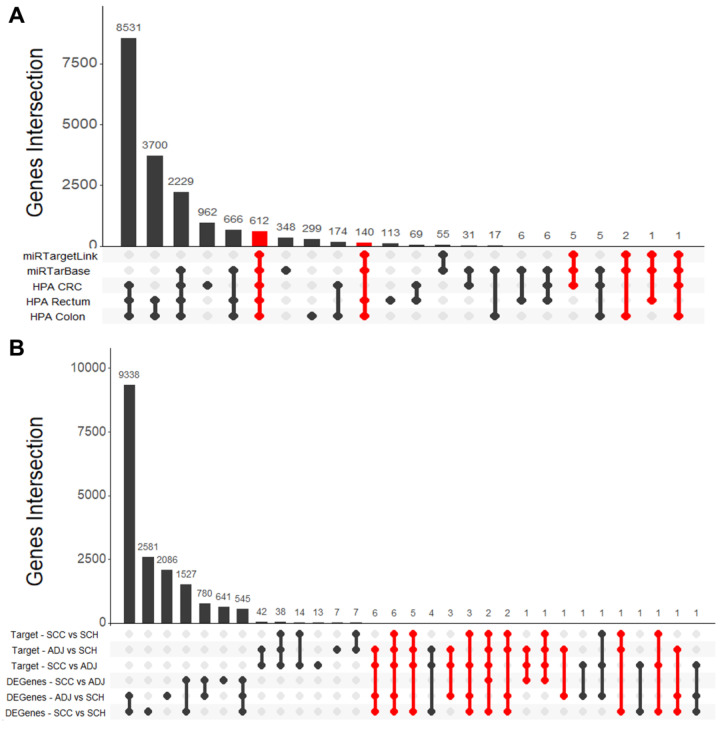
Upset plot for the select target genes to perform (**A**) gene enrichment and (**B**) an integrative analysis. Firstly (**A**), we selected target genes expressed in the healthy colon (HPA Rectum and HPA Colon) and CRC (HPA CRC), related to Human Protein Atlas databases. These genes were used to perform gene enrichment analysis. Then (**B**), we selected a gene subset to perform integrative analysis. For this, we considered only the genes that were involved with 23 cancer-related pathways subsets and were differentially expressed using TCGA and GTEx dataset. Highlighted in red are the genes selected to perform this analysis. The UpSetR package v.1.4.0 was used to create the plot.

**Figure 7:**
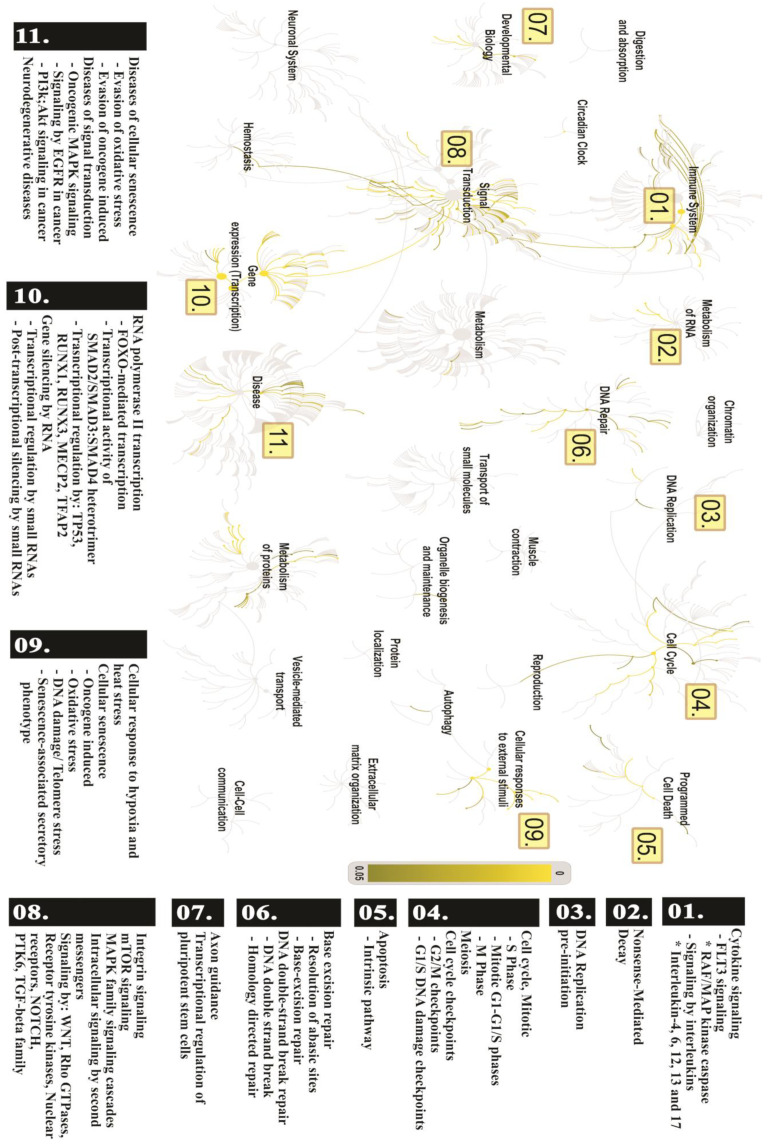
Genome-wide overview of the pathways analysis. Reactome pathways are arranged hierarchically. The center of each of the circular “bursts” is the root of one top-level pathway. Each step away from the center represents the next level above in the pathway hierarchy. The color code denotes the over-representation of that pathway in the input dataset. A numeric ID identifies the biological process significantly over-represented and correlated with a carcinogenic process (01–11), used to discriminate the significant main pathway involved in this process. Light grey represents pathways that are not significantly over-represented.

To better understand which pathways might be associated with the DEmiRNAs, we performed a functional analysis of its target genes. We found 816 target genes in common between miRTarBase and miRTargetLink Human. From those, 761 were selected for further investigation since they were expressed in CRC and healthy colon according to the HPA database (Figure 6A). Overall, these genes are involved in several cancer-related processes, such as inflammation, DNA repair, cell cycle, and programmed cell death (Figure 7).

**Figure 8:**
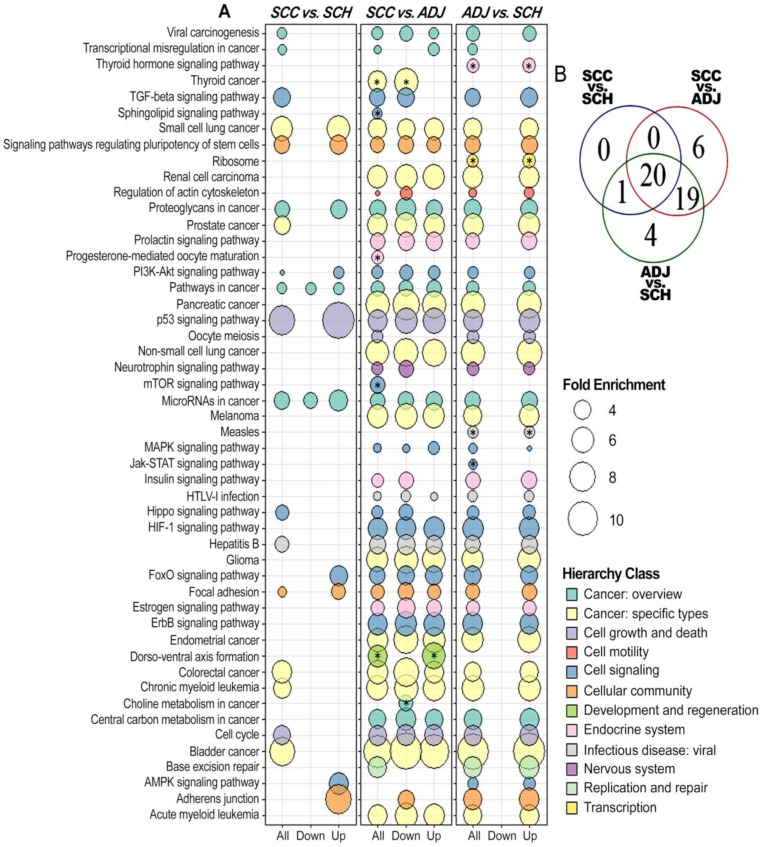
Gene set enrichment analysis of the DEmiRNAs. (**A**) The bubble chart shows significant pathways associated with the respective miRNA profile, according to the statistical model analyzed. This analysis was performed separately according to the profile of differentially expressed miRNAs. Fold enrichment was represented only for pathways that had Benjamin Hochberg’s adjusted *p*-value lower than 0.05. Each color represents a hierarchical class, according to the KEGG database. * = The KEGG was associated with one statistical comparison. (**B**) The Venn diagram shows all possible logical relations between the pathways obtained during gene enrichment analysis of statistical model sets.

Furthermore, we also performed a functional gene set enrichment analysis by each comparison (SCC vs. SCH; ADJ vs. SCH and SCC vs. ADJ), resulting in 50 enriched pathways (Figure 8). In the first comparison (SCC vs. SCH), we found that the p53 signaling pathway is strongly enriched, indicating that its four DEmiRNAs are related to cell growth and death. In the second comparison (SCC vs. ADJ), in addition to several cancer-related pathways, we also observed six pathways that are exclusive of this comparison (e.g., mTOR signaling pathway). In the last comparison (ADJ vs. SCH), we found four pathways restricted to this model (e.g., Jak-STAT signaling pathway) and also some cancer-related pathways.

Later, of these sets of 50 pathways, we selected a subset of 23 pathways that are cancer-related to explore. We searched for genes involved in each of these 23 pathways in TCGA (SCC and ADJ samples) and GTEx (SCH samples) and performed a differential expression analysis—data mining workflow is described in Figure 3. Using the same comparisons as before (SCC vs. SCH; ADJ vs. SCH and SCC vs. ADJ), we found 33 differentially expressed genes that are related to the subset of the 23 pathways (Figure 6B).

**Figure 9:**
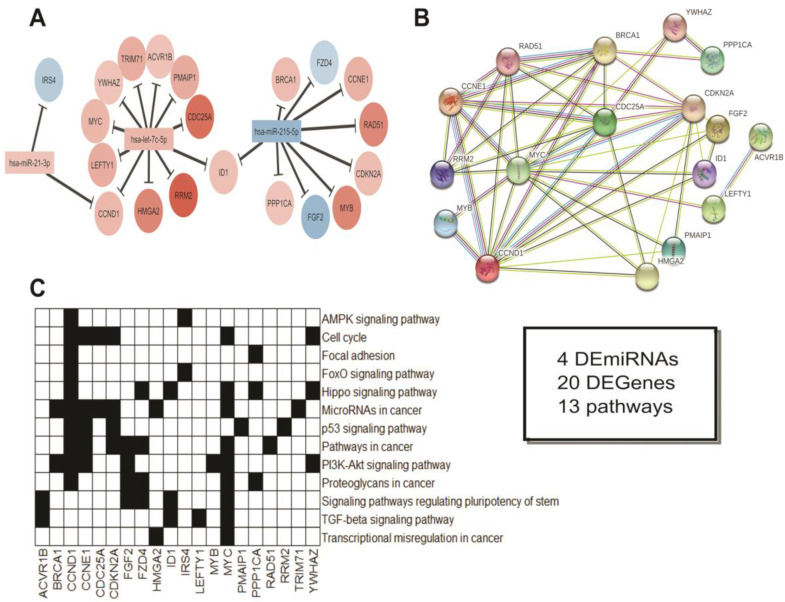
Integrative analysis of the DEmiRNAs in SCC vs. SCH. (**A**) miRNA-Gene and (**B**) Gene–Gene networks. In A, colors indicate if the miRNA or gene is up (red) or down (blue) expressed. miRNA Log_2_(Fold-Change) measures were obtained from our casuistic analysis, while gene Log_2_(Fold-Change) measures were obtained from TCGA and GTEx datasets. (**C**) Genes involved in the carcinogenic pathway, according to our functional annotation. The box indicates the total of miRNA and genes differentially expressed in this statistical comparison. Additionally, the total of pathways related to cancer was observed. Note: SCH = Sigmoid Colon Healthy. SCC = Sigmoid Colon Cancer. Disconnected nodes were hidden from the network view.

**Figure 10:**
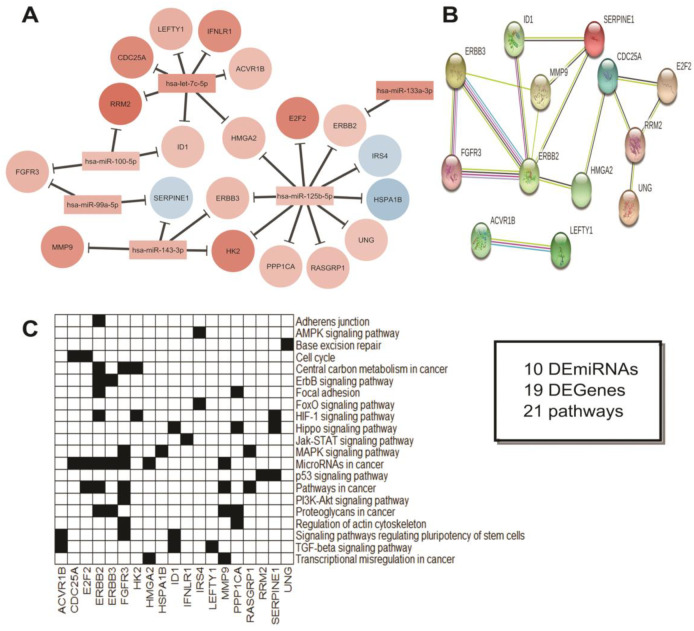
Integrative analysis of the DEmiRNAs in ADJ vs. SCH. (**A**) miRNA-Gene and (**B**) Gene-Gene network. In A, colors indicate if the miRNA or gene is up (red) or down (blue) expressed. miRNA Log_2_(Fold-Change) measures were obtained from our casuistic analysis, while gene Log_2_(Fold-Change) measures were obtained from TCGA and GTEx datasets. (**C**) Genes involved in the carcinogenic pathway, according to our functional enrichment analysis. The box indicates the total of miRNA and Genes differentially expressed in this statistical model. Additionally, the total of pathways related to cancer was observed. Note: SCH = Sigmoid Colon Healthy. ADJ = Adjacent-to-Tumor. Disconnected nodes were hidden from the network view.

**Figure 11:**
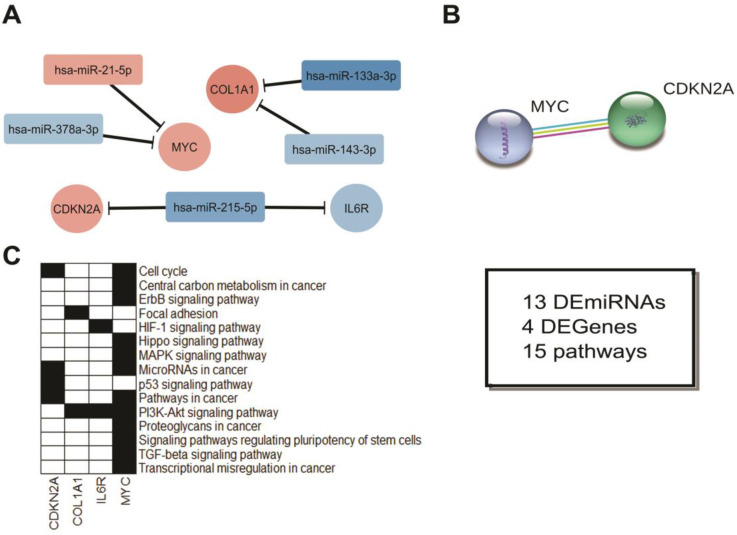
Integrative analysis of the DEmiRNAs in SCC vs. ADJ. (**A**) miRNA-Gene and (**B**) Gene-Gene network. In A, colors indicate if miRNA or gene is up (red) or down (blue) expressed. miRNA Log_2_(Fold-Change) were obtained from our casuistic analysis, while gene Log_2_(Fold-Change) were obtained from the TCGA dataset. (**C**) Genes involved in the carcinogenic pathway, according to our functional enrichment analysis. The box indicates the total of miRNA and genes differentially expressed in this statistical model. Additionally, the total of pathways related to cancer was observed. Note: SCC = Sigmoid Colon Cancer. ADJ = Adjacent-to-Tumor. Disconnected nodes were hidden from the network view.

Then, by using an integrative approach, we built three miRNA-gene regulatory networks involving these 33 genes and our 20 DEmiRNAs, according to our three comparisons — SCC vs. SCH (Figure 9), ADJ vs. SCH (Figure 10), and SCC vs. ADJ (Figure 11). We also performed a functional enrichment analysis related to each network. In the first comparison, we used four miRNAs and 20 genes and observed that *hsa-miR-let-7c-5p* and *hsa-miR-215-5p* might be involved in pathways related to cancer and cell cycle (Figure 9).

In the second comparison, we integrated ten miRNAs and 19 genes, which were mostly enriched for microRNAs in cancer, followed by pathways in cancer (Figure 10). These findings reinforce the notion that the adjacent tissue cannot be considered as normal as it presents several molecular alterations in comparison to the control group. However, the adjacent tissue cannot be considered as the tumor itself. The third comparison, which integrates four miRNAs and 13 genes, shows that there are some differences between these two types of tissues, being most of them related to the PIK3-Akt signaling pathway (Figure 11).

## 4. Discussion

In this study, we analyzed the differences in the microRNA expression profile of sigmoid colon, sigmoid colon cancer, and their counterpart. We also investigated the pathways in which the differentially expressed miRNAs are mainly associated. Furthermore, we constructed a miRNA-gene regulatory network based on the closely related target genes and miRNAs to better clarify the molecular mechanism of sigmoid colon cancer.

First, our data showed that the three types of colon tissue could be distinguished using a panel of 20 DEmiRNAs (Figure 4 and Figure 5). We accessed miRNAs’ global expression data from TCGA to further validate our DEmiRNAs of SCC vs. ADJ comparison and found similar results, corroborating our findings (Figure 4B). Then, we compared our 20 miRNAs-panel with previous studies [30,41,42,43,44,45,46,47,48,49,50,51,52,53,54,55,56,57,58,59], and observed that most of them had been reported in association with CRC, except for *hsa-miR-125b-2-3p*, *hsa-miR-1248*, *hsa-miR-190a-5p*, and *hsa-miR-424-5p* (Figure 4C), suggesting these miRNAs as potential novel biomarkers.

Our results showed that *hsa-miR-125b* is highly expressed in ADJ, when compared with SCH and SCC (Figure 4B), suggesting a potential activation of an initial carcinogenesis process, by inhibiting apoptosis [60] and promoting tumor proliferation [61]. On the other hand, *hsa-miR-1248* is predicted to regulate numerous cytokines, including IL-5 [62], facilitating metastasis colonization by modulating the immune-microenvironment [63]. The low expression of this miRNA in ADJ tissue, when compared with the healthy one (Figure 4B), suggests potential immune cell recruitment in response to inflammation triggered by the tumor [64].

**Figure 12:**
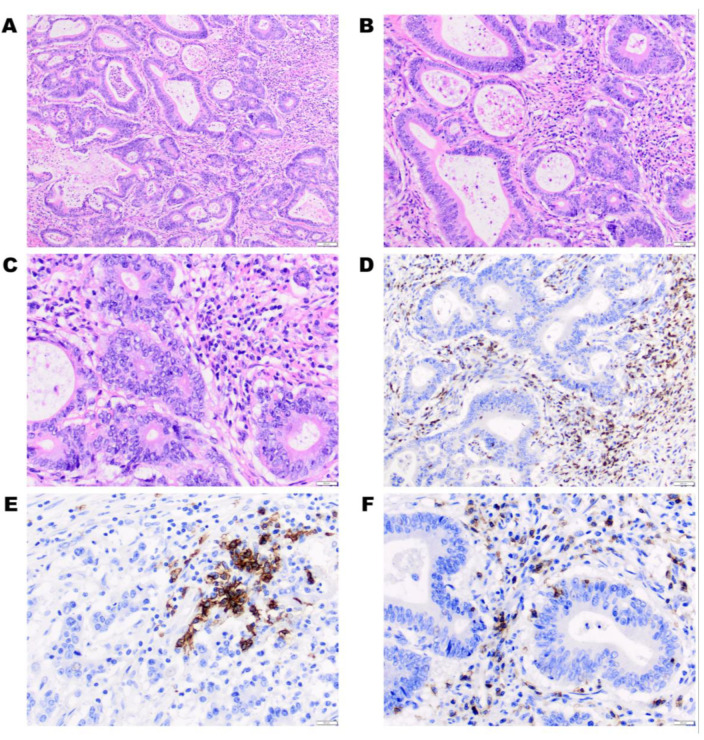
Histopathologic representation of tumor-infiltrating lymphocytes observed in the primary tumor. Hematoxylin and eosin stain, (**A**) ×100; (**B**) ×200; and (**C**) ×400. The following representative fields (**D**–**F**) show immunohistochemistry stain used to identify lymphocytes type. (**D**) The CD3+ indices presence of T lymphocytes, ×200. (**E**) The CD20+ marker indices presence of B lymphocytes, ×400; (**F**) the CD8+ marker indices presences of cytotoxic T lymphocytes, ×400.

Although the inflammatory mechanisms are essential for normal tissue regeneration [65], in the carcinogenic context, cytotoxic immune cells recognize and eliminate immunogenic cancer cells [66]. Meanwhile, they select cancer cell variants which are less immunogenic [66]. As it promotes tissue repair, failure of this can lead to persistent cytokine production, aggravating tissue destruction (such as necrosis) [67]. These findings corroborate with the histological data, which revealed that all patients had inflammatory cell infiltrates in the primary tumor (Table 1, Figure 12).

Downregulation of *hsa-miR-190a-5p* has been associated with fast-growing, clinically features, and potentially lethal cancer [68,69]. Moreover, its expression levels decrease with advanced tumor grade [68]. Our results showed that this miRNA had lower expression levels in tumor and adjacent-to-tumor tissue when compared to a healthy one (Figure 4B), suggesting a chronic carcinogenic process, which might be associated with tumor recurrence.

Interestingly, the role of *hsa-miR-424-5p* in the carcinogenesis process is controversial, depending on the cellular context [70]. It was already described as a tumor-suppressor in the esophageal squamous cell [71], hepatocellular [72], and ovarian tumors [73], while promoting cellular proliferation in other tumors, such as gastric [74] and pancreatic cancer [75]. Our results showed a higher expression in the cancer when compared with its counterpart (Figure 4B), which indicates that this miRNA might be related to *TGFBR3* [70]. This can result in the disruption of a pathway commonly associated with colon cancer, the TGF-B superfamily signaling [76].

The global functional enrichment of the 20 differentially expressed miRNAs revealed genes involved in several processes altered in cancer, including cell cycle [77] and death [78,79] (Figure 7). Additionally, the p53 signaling pathway was the most enriched process in the comparison between healthy and cancer samples. This shows the significant impact of these miRNAs in carcinogenic pathways, by controlling most of the cancer hallmarks—such as deregulated proliferation and cell death, replicative immortality, angiogenesis, invasion and metastasis, metabolism and genomic instability, as well as immune response (Figure 8). The *hsa-miR-let-7c-5p* and *hsa-miR-215-5p* were identified regulating most of the differentially expressed genes found between SCC and SCH (Figure 4) and were closely related to the carcinogenic pathway, regulating the expression of *RRM2*, *PMAIP1*, *CDKN2A*, *CCNE1*, and *CCND1* (Figure 9).

The let-7 family has been described as a tumor-suppressor, repressing several oncogenes, influencing cell proliferation and apoptotic pathways [80]. The upregulation of certain let-7 family members has also been observed with less frequency, suggesting that *hsa-let-7* does not act as a tumor-suppressor under all circumstances or tissues [81]. Its upregulated expression has been associated with cell differentiation [80], indicating that the increased expression could be used as a prognostic marker to identify patients at risk. In hepatocellular carcinoma, the upregulation of this miRNA was associated with high-grade tumors [80]. This finding corroborates our histological data, which show that all patients had an intermediate-grade tumor (Table 1). Concerning *hsa-miR-215-5p*, it has been reported in the literature as being downregulated in tumor tissue, as shown in Figure 4C. Its expression reduces according to clinical stage progression and the presence of lymph node metastases [46], enabling tumor proliferation and migration [82,83].

The regulatory network analysis, presented in Figure 10A, revealed evidence of two hub miRNAs in the comparison of ADJ and SCH: *hsa-miR-let-7c-5p* and *hsa-miR-125b-5p*. The *hsa-let-7c-5p* demonstrated the same aberrant expression profile in adjacent-to-tumor and tumor tissue when compared with healthy tissues (Figure 4B). This finding suggests that the adjacent-tissue might be in the malignant transformation process, by impact in immune-response [84,85] or already had a compromised cell differentiation, as discussed above. Concerning *hsa-miR-125b-5p*, its role in carcinogenesis is unclear, as it could be acting either as a tumor suppressor or an oncogene, as reviewed by Sun et al. [61]. The ectopic expression of this miRNA in adjacent-to-tumor tissue, when compared with the healthy one, suggests several possibilities of its role in recurrence and tumor progression. First, *hsa-miR-125b* might be suppressing the ERBB2 and ERBB3 pathway, reducing proliferative growth, motility, and invasiveness of tumors [86]. Second, elevated expression of *hsa-miR-125b* might be driving macrophages to an active form, accompanied by increased co-stimulatory factor expression and elevated responsiveness to interferon-gamma [87]. Furthermore, high macrophage infiltration has been associated with both better and worst prognosis according to immune microenvironment stimuli [88].

Regarding the integrative network made for SCC and ADJ comparison, we found four hub miRNAs (Figure 11A): *hsa-miR-215-5p*, *hsa-miR-378a-3p*, *hsa-miR-133a-3p*, and *hsa-miR-143-3p*. Similarly, when compared with the healthy tissue, *hsa-miR-215-5p* presented as downregulated in tumor tissue (Figure 4B), reinforcing its role in clinical-stage progression, as discussed above. The *hsa-miR-378a-3p* profile showed to be consistent in different studies (Figure 4C), corroborating our findings, indicating its role in inhibiting apoptosis, and promoting tumor proliferation [89]. Furthermore, its expression might be reduced as a result of an inflammatory process occurring in the tissue [90]. Regarding *hsa-miR-133a-3p* [91] and *hsa-miR-143-3p* [92], they might modulate epithelial–mesenchymal transition, impacting cell adherence and tight junction, conferring an invasive phenotype to various cancers. Additionally, as shown in Figure 11C, these miRNAs are mainly involved in the PI3K-Akt signaling pathway, suggesting the impact of this pathway in differentiating the tumor itself from the adjacent-tumor samples. This impact might be acting in synergy with adherence and tight junction aberrations [93], consolidating the association of these miRNAs in malignant transformation and suggesting them as potential biomarkers to evaluate safety margins or stratified potential of recurrence cases.

Our results show that although adjacent-to-tumor tissues have unique characteristics differentiating them from both healthy and tumor tissues (Figure 4A and Figure 5B), they also share a few components, such as *hsa-miR-215-5p* and *hsa-let-7c-5p*, respectively. In line with these findings, we observed that both the tumor and adjacent tissues share pathways that might be compromised—such as p53 signaling pathways [94], the Hippo signaling pathway [95,96], AMPK signaling pathway [97], and FoxO signaling pathway [98]. Furthermore, we could observe that DEmiRNAs in tumor tissue can influence pathways such as Focal adhesion [99], the PI3K-Akt signaling pathway [100], and TGF-beta signaling pathway [101], influencing on the adjacent-tissue molecular profile [102].

To date, there are limitations regarding sample number, lack of available data, and lack of prior research studies using healthy tissue as control. Several studies have used adjacent-to-tumor tissue as controls in their approaches, reasonably by having an advantage of easy sample access. However, by comparing only tumor tissues with their counterpart, many potential cancer biomarker candidates may be missed, and others spuriously implicated [103,104], as exposed above.

The present study reinforces the importance of future studies considering the field-effect theory during method design, as well as previous works published by our group in studies involving other cancers [23,26,27]. Our study also raises several opportunities for future research, both in terms of theory development and concept validation. We believe that some questions could be elucidated if we also use healthy tissue as a control, for example: How could the adjacent tissue impact carcinogenesis? What are the molecular differences between tumor and adjacent-to-tumor concerning healthy tissue, according to the TNM stage? How could stromal molecular components influence the tumor progression? In which moment could the adjacent tissue turn into a cancer, be it second primary tumor or local recurrence? How could we avoid this malignant process? What are the best treatment strategies according to the molecular profile? Moreover, we believe that new therapeutic targets could be identified as an improvement in therapy against cancer.

## 5. Conclusions

In summary, our results demonstrated that 20 DEmiRNAs could distinguish the three types of tissue evaluated here (SCC, SCH, and ADJ). Additionally, we found enriched pathways related to the carcinogenic process in which these miRNAs could be involved, indicating that the adjacent-to-tumor tissue may already be molecularly altered; therefore, it cannot be considered as healthy. Hence, it is also essential to include healthy non-cancer tissues in case-control studies, since it may give a better understanding of the molecular alterations occurring during the malignant transformation. Overall, we expect that these findings may help in the search for biomarkers to prevent cancer development allowing its early detection. A further validation may confirm this applicabilities. Moreover, we strongly recommend that future cancer studies consider using these three tissues in their analysis, as well as we suggest the creation of a database of healthy individuals’ miRNA expression profiles.

## Figures and Tables

**Table 1:** Clinical characteristics of patients at diagnosis.

Characteristics	Cases (*n* = 7)	Control (*n* = 7)
Age	60.86 ± 10.4 years	51.85 ± 17.44 years
Sex (M/F)	5 (71.43%)/2 (28.57%)	3 (42.86%)/4 (57.14%)
Tumor grade (Intermediate)	7 (100%)	-
Histological classification		
Non-mucinous adenocarcinoma	6 (85.71%)	-
Mucinous and non-mucinous adenocarcinoma	1 (14.29%)	-
Inflammatory cell infiltrates (Yes)	7 (100%)	-
Tumor budding		
Yes	1 (14.29%)	-
No/Not informed	5 (71.43%)/1 (14.29%)	-
Depth of invasion		
T1, T2	2 (28.57%)	-
T3, T4	4 (57.14%)	-
Tx	1 (14.29%)	-
Lymph node involvement		
N0/Nx	4 (57.14%)/1 (14.29%)	-
N1, N2	2 (28.57%)	-
Distant metastasis		
M0/Mx	5 (71.43%)/1 (14.29%)	-
M1	1 (14.29%)	-
AJCC stage		
Stage I/Stage II	2 (28.57%)/1 (14.29%)	-
Stage III/Stage IV	2 (28.57%)/1 (14.29%)	-
Unknown	1 (14.29%)	-
Metastasis site (Liver/Not identified)	1 (14.29%)/6 (85.71%)	-

Note: Tumors were classified according to the American Joint Committee on Cancer (AJCC) staging system.

**Table 2:** Clinical characteristics of patients selected from the GTEx and TCGA dataset.

Characteristics	TCGA—miRNA-Seq (*n* = 3)	TCGA—RNA-Seq (*n* = 13)	GTEx (*n* = 173)
Age	62.66 ± 20.42 years	64.69 ± 14.51 years	Not informed
Sex (M/F)	33.33%/66.67%	53.85%/46.15%	59.24%/40.46%
AJCC stage			
Stage I/Stage II	100%	84.62%	-
Stage III/Stage IV	-	15.38%	-
Unknown	-	-	-

Note: Tumors were classified according to the guidelines of the American Joint Committee on Cancer (AJCC) staging system.
